# Challenges and opportunities: computational biology and the future of agriculture

**DOI:** 10.1093/bioadv/vbag003

**Published:** 2026-02-03

**Authors:** Joao Carlos Gomes-Neto, Alexandra Crook, Rachel Hestrin, Guoming Li, Chia-Sin Liew, Guilherme Rosa, Keshav D Singh, Christopher K Tuggle, Katie L Summers, Camilo Valdes, Noah Fahlgren, Jennifer Clarke

**Affiliations:** Department of Animal Science, Ohio State University, Wooster, OH 44691, United States; Center for Food Animal Health, Ohio State University, Wooster, OH 44691, United States; Department of Biochemistry and Molecular Genetics, Anschutz Medical Campus, University of Colorado, Aurora, CO 80045, United States; Stockbridge School of Agriculture, University of Massachusetts Amherst, Amherst, MA 01003, United States; Department of Poultry Science, The University of Georgia, Athens, GA 30602, United States; Center for Biotechnology, University of Nebraska-Lincoln, Lincoln, NE 68588, United States; Department of Animal and Dairy Sciences, University of Wisconsin-Madison, Madison, WI 53706, United States; Agriculture and Agri-Food Canada (AAFC), Lethbridge, AB T1J4B1, Canada; Department of Animal Science, Iowa State University, Ames, IA 50011, United States; Animal Biosciences & Biotechnology Laboratory, USDA, Beltsville, MD 20705, United States; Physical and Life Sciences Directorate, Lawrence Livermore National Laboratory, Livermore, CA 94551, United States; Data Science Facility, Donald Danforth Plant Sciences Center, St Louis, MO 63132, United States; Department of Statistics, University of Nebraska-Lincoln, Lincoln, NE 68583, United States; Department of Food Science and Technology, University of Nebraska-Lincoln, Lincoln, NE 68588, United States

## Abstract

**Motivation:**

The world of agriculture is rapidly changing with advances in artificial intelligence and demands for greater feed and food security considering environmental and sustainability challenges. The 30th Conference on Intelligent Systems in Molecular Biology (ISMB) held in July 2022 featured an invited session on the role of computational biology in Digital and Precision Agriculture. This session featured presentations by experts from various subdisciplines on novel research discoveries and a panel discussion on Digital Agriculture at Scale. Topics discussed during the session included genetics, epigenetics, and genomics of agriculturally relevant species; foodborne pathogen genomics and epidemiology; plant and animal phenomics; AI/machine learning; image analysis; remote sensing; educational innovations; discoveries resulting from public-private partnerships; data sharing and findable, accessible, interoperable, and reproducible (FAIR) data standards; biotechnology; and soil microbial ecology and biogeochemistry.

**Results:**

We present several of the current and future challenges and opportunities for computational biology in agriculture including why these challenges are important to address, what barriers exist, and what skills and competencies are required to be successful as a computational biologist in agriculture. We intend this summary to engage the computational biology community and attract them to the opportunities available for interesting and impactful work toward ensuring sustainable food security.

## 1 Introduction

A societal grand challenge for the 21st century is providing a robust, safe, and nutritious food supply when global food demand is expected to increase by 35% to 56% between 2010 and 2050 (van Dijk *et al.* 2021). The Digital and Precision Agriculture track at the 30th Annual Meeting of the International Society for Computational Biology (ISCB) invited abstracts for research that was topical to the field of digital agriculture (DA) from basic to applied sciences including intersections with other disciplines. Topics relevant to this Special Session included, but were not limited to, genetics and genomics of agriculturally relevant species; foodborne pathogen genomics and epidemiology (food safety); plant and animal phenomics; artificial intelligence/machine learning (AI/ML); data sharing and findable, accessible, interoperable, and reproducible (FAIR) data standards; biotechnology; and soil microbial ecology and biogeochemistry. Digital Agriculture (DA) is broadly defined as practices that facilitate the transformation of farming through automatization of tasks and implementation of biotechnology to improve food safety and productivity across crops and livestock production systems (Rajak *et al.* 2023). DA can be applied to a multitude of areas that directly impact crop and livestock productivity and profitability on a scalable level to meet market demands. For instance, DA has enhanced and transformed the crop planting and processing chain by deploying automation, sensors and machinery capable of multiple tasks while utilizing novel AI/ML approaches that allow for large scale predictive modeling (Crook *et al.* 2021, Hoyos-Villegas *et al.* 2025). DA and computational biology can be used to monitor soil characteristics, predict soil microbial activity, and enable microbiome-based interventions that support more resilient agricultural production systems (Hestrin *et al.* 2022, Rajak *et al.* 2023). As for livestock, although there remain challenges for scalability and the range of application varies between species, there have been notable successes in automated feeding and animal management, medication/vaccination strategies, behavioral analysis, welfare assessment, animal breeding and selection including establishing public resources for functional annotation of genetic variants, along with surveillance of pathogens through genomics (Arfken *et al.* 2020, Chaudhari *et al.* 2021). This perspectives article combines suggestions from scientists working across crop and livestock agricultural sciences regarding challenges and opportunities for the ISMB community in DA. We conclude with a summary of actionable priorities in Table 1.

## 2 Research challenges

Due to the complexity of agricultural production systems worldwide, productivity and profitability are multifactorial metrics with many inputs/outputs and high variance. We recognize that the integration of biological data sets relevant to microbiomes, host genetics, phenotypes, narrow and broad gene expression, along with epidemiological/environmental data, presents a significant challenge for DA. Nonetheless, if digitization and computational biology are to enhance the efficacy of agriculture in major production centers worldwide, we will need to address critical issues that hinder deployment, adoption and cost-effectiveness. We succinctly describe some of the topics from our invited session that highlight the key role of computational biology in agricultural research.

### 2.1 Genomics

Advances in genomics are critical to several aspects of agriculture. An essential need is improved annotation and curation of databases for agriculturally relevant pests, pathogens, plants, beneficial microbes, and animals. Several excellent genome assemblies with annotations exist (see, e.g. https://www.agbiodata.org/databases/). However, maintaining and ensuring their consistency and quality remains a significant challenge. This is especially true for plant genomes due to their complexity, large sizes, variable ploidy, the presence of transposable elements, and abundant repetitive regions (Freedman and Sackton 2025). Benchmarking efforts have been made to come up with best practices depending on the datasets and target species (Park *et al.* 2023) and a conceptual framework based on LLMs has been proposed (Li *et al.* 2025). One example is fungal database curation complications such as widespread misidentification of genomic sequences, taxonomic complexities, and significant gaps in data. These severely limit accurate fungal research and identification (Lücking *et al.* 2021) which is critical to using genomics to develop novel biocontrol agents, biofuel, food and food additives. Another example is deploying new algorithms that maximize the use of the Pathogen Detection system at NCBI (The NCBI Pathogen Detection Project 2016) for epidemiological and ecological inquiries. The NCBI system can cluster related pathogen genome sequences to identify potential transmission chains and screen sequences for anti-microbial resistance and virulence genes (Feldgarden *et al.* 2022). Unfortunately, it relies on a phylogenetic clustering methodology that inhibits real-time scalability and mapping of novel mutations and loci that may differentiate emerging strains. The development and integration of new bioinformatics tools that leverage long-read sequencing, integrate functional information, and enable host-response modeling would enhance the existing system (National Academies of Sciences, Engineering, and Medicine 2025). An additional example is pangenomic resources in crop breeding. Recent pangenome projects in maize (Schreiber *et al.* 2024) and other crop species have revealed structural variants linked to kernel size, drought tolerance, and leaf architecture. Computational biology is essential to these projects with graph-based genome assembly and structural variant calling algorithms enabling breeders to capture genetic diversity beyond reference genomes.

### 2.2 Phenotyping

Similar challenges and needs exist in phenomics. In part this is due to the variety of the applications and data types involved in phenotyping (Callwood *et al.* 2025). There are needs for improved algorithms and tools for image-based analyses of soil, field, livestock, and crops for high-throughput collection of data as well as spatial and temporal collection and analyses of data for ecologically oriented decision making (e.g. wineries). An example is the effort to map the global distribution of soil biota (Delgado-Baquerizo *et al.* 2018), link environmental conditions to microbial metagenomes, create novel tools for microbiome data (Valdes *et al.* 2023), and develop open-access databases and computational tools (such as the Department of Energy’s National Microbiome Data Collaborative and Systems Biology Knowledgebase). This effort rests heavily on computational biology expertise. Yet there remains a substantial gap between big data-based approaches and actionable recommendations for stakeholders who seek to manage agricultural microbiomes for more efficient and profitable production.

## 3 The importance of computational biology in agricultural research

The topics mentioned above can be characterized as opportunities for computational biologists to improve sustainable agricultural productivity and profitability. By identifying these opportunities, we can map what are the auxiliary areas that should be encompassed in the development of DA. For instance, the enhancement and scaling of the use of genomics depends on data storage, improved gene annotation and curation of sustained databases, which in turn requires more robust and optimized algorithms, ultimately necessitating the training of professionals and scientists in computational biology. Therefore, we briefly describe the importance of several challenges being addressed by DA and computational biology.

### 3.1 Livestock

Advances in selection and improvement of animal populations is critical to food security. Examples are improvement of dietary efficiency through gastrointestinal microbiome modulation; improvement in prediction of biological outcomes from genetic variation filtered for predicted functional impact on phenotype; and rapid disease diagnosis based on animal traits such as scours, temperature, and/or feed intake. Readers are referred to Koltes *et al.* (2019) for one vision of using large-scale data analytics to address the use of high-throughput data types in livestock genetic improvement.

### 3.2 Agronomy

**Figure 1:**
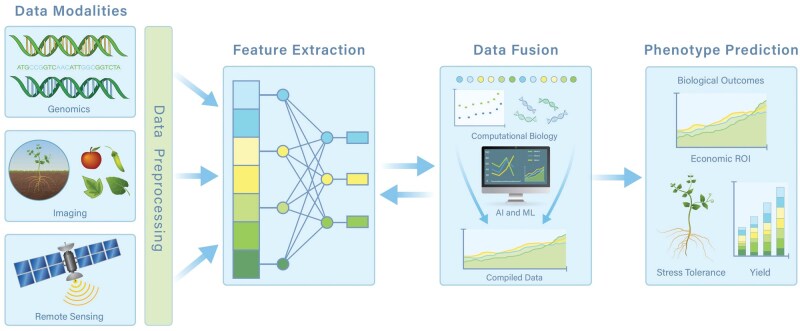
Example of computational biology in agricultural research. Note that yield prediction is only one of many possible outcomes, e.g. prediction of stress resistance in livestock or crops. Adopted from Ferreira *et al.* (2024).

Current agronomic practices, particularly cropping systems, have been optimized for resource-rich, stable environments and climate conditions of the 20^th^ century. Future agricultural productivity rests on improving our understanding of the impact of spatial/temporal and other environmental factors ion yield and stress resilience while accounting for ecosystem changes and limited resources. This involves linking plant genetics and molecular biology with phenotypic traits including yield and abiotic and biotic stress resistance (Tuggle *et al.* 2024). The translation of findings from system and computational biology to agronomic practice relies on implementation of high-throughput phenotyping for real-time prediction of yield, disease resistance, and accurate estimation of management impacts (see Fig. 1).

### 3.3 Microbial pathogens

The COVID pandemic was a stark reminder of the importance of molecular epidemiology and surveillance of viral and bacterial pathogens at a global level in real-time for decision-making. There is an ongoing need for the development and updating of vaccines based on population-based immunological epitopes as well as screening for novel drugs against viral and bacterial pathogens. Ongoing microbiome research is using advanced computational biology expertise to discover microbiome-based traits (taxa) that confer colonization resistance against pathogens in a host associated fashion. This research may apply not only to humans but to agronomically important species (Pavlovikj *et al.* 2021). Advances in molecular diagnostics could result from novel methods in computational biology that accurately predict anti-microbial resistance (AMR) and/or disinfectant resistance in foodborne pathogens solely based on population structure using linkage disequilibrium among loci.

**Figure 2:**
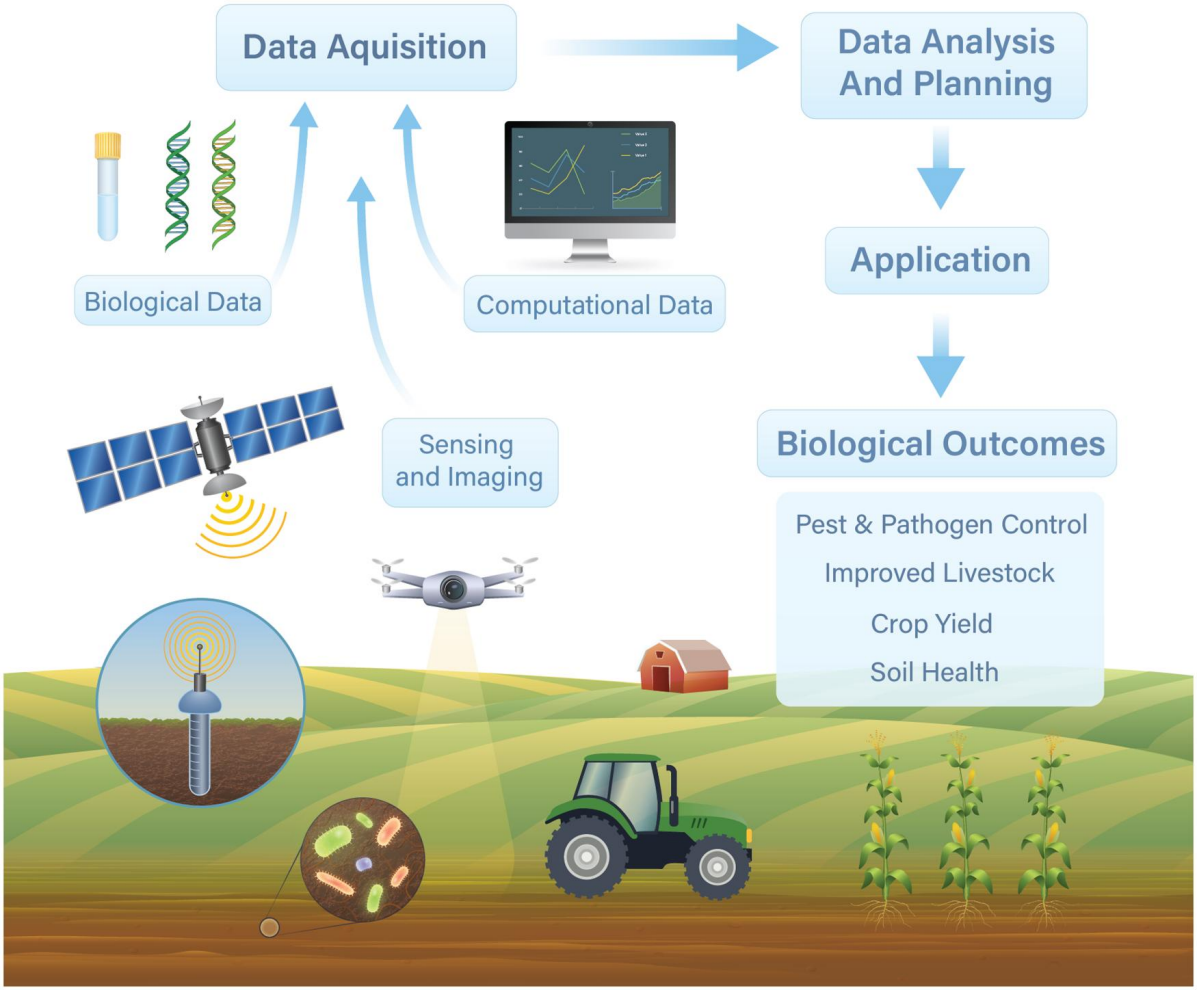
Conceptual framework for integration of computational biology in agricultural research. Each data type, including biological, may be of several different modalities, further complicating both collection and analysis.

Figure 2 presents a conceptual figure for how computational biologists can play critical roles in agricultural research. There is an urgent need for expertise in integration of biological data with computational data (e.g. machinery data, management data) and remote sensing and imaging data to improve important agricultural outcomes. The biological data may be of multiple types (e.g. omics, laboratory testing, bioimaging) and both collection and integration would benefit from computational biologists.

### 3.4 Methodology/ethics

Any discussion of the importance of computational and data-driven research must mention the critical role of FAIR and CARE standards. The impact and value of collaborations between computational biology and agriculture rests on adhering to these standards as they are critical to maintaining the trust of the agricultural community at all levels (including academia, industry, and regulatory agencies). This trust underlies access to data and critical use cases which translate into impact. As such, what is ethical or moral in this domain is a shared decision among researchers, farmers, ranchers, and stakeholders. We rely on Institutional Review Boards and Responsible Conduct of Research institutional policies to guide us while remaining cognizant of the expectations of agricultural practitioners. We further expect that documentation of such standards will be provided by authors (and verified by publishers) before publication of research and resource development results. One suggestion is that such a document be developed by the Food and Agricultural Organization of the United Nations (FAO).

## 4 Bottlenecks that must be addressed to help overcoming these research challenges

The DA challenges presented here require several bottlenecks to be addressed for the successful implementation of solutions. These bottlenecks represent professional opportunities for computational biologists as well as agricultural researchers.

### 4.1 Human resources

There is a need for broader access to quantitative training for biologists, animal scientists, agronomists including for professional degrees such as Veterinary Medicine. Some topics of critical need include biostatistics with some math background exposure, coding in R and Python and exposure to the R and Python libraries, command line training, learning to combine knowledge from observational and experimental datasets, data interpretation and reasoning, and data reporting and translation to stakeholders. Data driven computational skills could be incorporated into training programs very early in the curriculum, while a stronger quantitative foundation is being built-up. Of course, it is unreasonable to expect any one person to become an expert in everything. Therefore, we believe that novel teams must be formed as a consortium to enhance specific areas of research, along with industry level insights, for the discovery and implementation of practical solutions for both crops and livestock. From a professional research training perspective, we see the need for (1) flexible graduate level programs, including short-term credentials, with a strong quantitative, computational, and scientific training; (2) forming cross-disciplinary teams with specific research tasks in DA; (3) providing training in the area of research ethics and reproducibility; (4) bringing computational biology, biostatistics and coding expertise and pedagogy to training programs; and (5) looping in industry advisors who could help research programs in developing tools that solve practical problems.

### 4.2 Database collections and data federation

As mentioned above, there is an immediate and urgent need for more accurate and complete genomic data annotation, curation, and mapping at all levels and organisms with sustainable support. We think this could be optimized by developing organismal specific datasets that are curated by a core-team of computer scientists and researchers from multiple institutions. As a result, we believe this process could yield more specific grant application processes (e.g. informing grant agencies with data of the issue and help the development of such infrastructure). Geospatial specificities and needs must be addressed to make sure we democratize knowledge and technological advances. The current limitations in the agricultural research community for effective data reuse are described in Hafner *et al.* (2025).

### 4.3 Algorithm and software development

There is a need for problem-oriented algorithm development that uses a bottom-up approach to decompose the problem and develop tools for it, instead of applying packages that are limited in portability. For instance, if trait prediction from genomic data is the goal, a theme that emerged from the talks was that the incorporation not only of genomic and phenotyping data is needed for the model, but also economic value, and ecological factors that may shape model accuracy. As such, we can envision the development of novel software linked to a host-specific database, capable of integrating layers of information, while updating the models with real-time data that can be influenced by market volatility and geographic influences.

### 4.4 Computational

The cost and effectiveness of using computational tools is a major obstacle. Not all institutions and partners can run large scale dataset analyses on demand and affordable and accessible data storage is limited, particularly for proprietary or identifiable data. An opportunity we see is the development of multi-institutional computational infrastructure combining the knowhow of scientists. Efforts like the National Artificial Intelligence Research Resource (NAIRR) launched by NSF and AI-on-Demand (AI4Europe) supported by the European Union are steps in the right direction. Alternatively, institutions could partner with the private sector to buy computational power as needed and have the advantage of using cutting-edge hardware technology. Multi-sector partnerships are crucial as cloud infrastructure is the cornerstone for the development of complex and large-scale projects across disciplines.

**Table 1:** Summary of actionable priorities.

Human resources: data and computational training in agriculture, and vice versa, paired with domain expertise
Public private partnerships for research, education, and translation
Interdisciplinary team development integrating data and agricultural sciences and industry partners
Funding opportunities for tool and database development with careful data curation and experimental validation

### 4.5 Financial support

Last but certainly not least, we need to secure the resources required to advance research and training in computational biology and agriculture, as well as the translation of discoveries to practice. There are several types of financial support that could provide an optimal environment for this effort. We include here short-term grants that focus on answering a specific research hypothesis or training goal, as well as longer-term, infrastructure-based support and resources that the community can use to build collaborative research and training projects. Possible avenues for funding include NSF National Research Traineeships, USDA ARS and NIFA grants; Horizon Europe 2028-2034; and non-governmental organizations. We also want to prioritize funding from industry through public-private partnerships and international consortia.

## Data Availability

No data to report.
